# Low doses of eriocitrin attenuate metabolic impairment of glucose and lipids in ongoing obesogenic diet in mice

**DOI:** 10.1017/jns.2020.52

**Published:** 2020-12-14

**Authors:** P. S. Ferreira, J. A. Manthey, M. S. Nery, L. C. Spolidorio, T. B. Cesar

**Affiliations:** 1 Laboratory of Nutrition, School of Pharmaceutical Sciences, Sao Paulo State University – UNESP, Araraquara 14800-903, SP, Brazil; 2 U.S. Horticultural Research Laboratory, Agricultural Research Service, USDA, 1401 South Rock Road/Port Fierce, FL 34945, USA; 3 Laboratory of Pathology, School of Dentistry, Sao Paulo State University - UNESP, Araraquara 14801-385, SP, Brazil

**Keywords:** Eriocitrin, Obesity, Metabolism, Systemic inflammation, Oxidative stress

## Abstract

Eriocitrin is a citrus flavonoid with a high capacity to reduce the oxidative stress related to metabolic disorders and obesity. We assessed the effects of low doses of eriocitrin on the oxidative stress, inflammation, and metabolism of glucose and lipids of high-fat diet (HFD)-fed obese mice. Fifty male C57BL/6J mice were randomly assigned into five groups (*n* 10). The mice were fed an HFD (45 % kcal from fat, i.e. lard) for 4 weeks for obesity induction. After this period, the mice continued receiving the same HFD, but supplemented with eriocitrin at 10, 25 or 100 mg/kg body weight (bw) for an additional 4 weeks. Control groups were fed with standard diet (10 % kcal of fat, i.e. soy oil) or with HFD without eriocitrin, for eight consecutive weeks. At the end of the study, mice supplemented with eriocitrin showed lower levels of blood serum glucose and blood and liver triacylglycerols (*P* < 0⋅05). There was also improved levels of insulin, HOMA-IR, total-cholesterol, resistin and lipid peroxidation in the supplemented mice. It was concluded that the 25 mg dose of eriocitrin improved all the parameters studied and had positive effects on oxidative stress, systemic inflammation and metabolism of lipids and glucose in general.

## Introduction

Human obesity-related metabolic dysregulation can be modelled in mice by the ingestion of high-fat diets (HFDs)^(1–3)^, which increases free radical production pathways in the liver and adipose tissue, stimulates the production of pro-inflammatory molecules and acute-phase proteins, and as well as elevates the level of blood lipids^(4,5)^. Excess circulating triacylglycerols lead to systemic lipotoxicity, which then dysregulates cardiac and hepatic functions and the activity of insulin, mainly in the heart, liver and muscles. In addition, this abdominal obesity releases more free fatty acids and cytokines, which contribute to the perpetuation of damaging systemic inflammation^(6,7)^.

Because the global rates of obesity nowadays and its related diseases, there is a great demand for natural ingredients capable of neutralising the deleterious effects of these conditions and preventing further complications. Citrus flavonoids have been shown to improve metabolic disorders linked to inflammation and oxidative stress in animal models^(8,9)^. Eriocitrin (eriodictyol-7-*O*-rutinoside) and its aglycon eriodictyol are the main flavanones isolated from lemon peel (*Citrus limon*)^(10)^. They have been recognised as antioxidant and anti-inflammatory agents, capable of reducing the levels of TNF-α, intercellular adhesion molecule 1 (ICAM-1), vascular endothelial growth factor (VEGF) and endothelial nitric oxide synthase in diabetic rats^(9)^. A previous study from our laboratory showed that both eriocitrin and eriodictyol prevented systemic inflammation and reduced oxidative stress markers in the liver and blood of obese mice^(11)^. In addition, eriodictyol was shown to contribute to the endogenous antioxidant system through the induction and preservation of antioxidant enzymes^(12–14)^ and, thus, to improve lipid and glucose metabolism^(15,16)^.

Eriocitrin metabolism begins in the colon, where it is converted to eriodictyol, absorbed into the enterocytes and conjugated with hydrophilic molecules to be transported to the liver through the portal circulation. In the liver, eriodictyol is metabolised again, generating conjugated metabolites of eriodictyol, homoeriodictyol and hesperetin, which return to the bloodstream ready to exert their biological activities^(17–19)^. It was shown that a lemon flavonoid extract containing 33 % eriocitrin could be safely administered up to 2 g/kg bw/day for 4 weeks^(20)^; however, there is no evidence on its safety in long-term administrations or on its effectiveness in much lower doses. Although earlier studies have reported beneficial effects of eriocitrin *in vivo*, most of the studies have not explored the efficacy of this flavonoid in counteracting obesity-induced metabolic disturbances at doses that would be practicable for human^(11,15,21)^. Thus, the present study is aimed at examining the effectiveness of the dietary supplementation of lower doses of eriocitrin on the adverse effects of HFD-induced obesity in C57BL/6J mice.

## Materials and methods

### Experimental design

Fifty male C57BL/6J SPF mice, 6-weeks old (20 ± 1 g), from the University of São Paulo, Ribeirao Preto, SP, Brazil, were kept in a HEPA-filtered ventilated cabinet with a constant temperature of 22–25°C and 12 h light/dark cycles. After 1 week of adaptation, the mice were randomly assigned into five groups of ten mice each using simple random sampling, being: (1) Standard group, the control group fed a standard diet containing 10 % of calories from fat (AIN-93M); (2) HFD group, fed an HFD containing 45 % calories from fat (lard); (3) HFD + eriocitrin10 mg/kg group, fed an HFD supplemented with 10 mg/kg of eriocitrin; (4) HFD + eriocitrin25 mg/kg group, fed HFD supplemented with 25 mg/kg of eriocitrin; and (5) HFD + eriocitrin100 mg/kg group, fed an HFD supplemented with 100 mg/kg of eriocitrin. The experimental period was divided into two periods of 4 weeks; during the first 4 weeks obesity was established by the use of the HFDs in the HFD groups, and the second 4-week period included the dietary supplementations with eriocitrin in the HFD. During the entire experimental period, mice had free access to autoclaved food and water. The weight gain was measured weekly and the food intake daily. Eriocitrin and energy intake were calculated based on daily food intake. This experimental protocol was approved by the Animal Use Committee of the São Paulo State University (UNESP), School of Pharmaceutical Sciences, Araraquara, SP, Brazil (16/2015), and follows Brazilian National Board for Animal Experimentation Control (CONCEA) guidelines.

### Supplementation

Eriocitrin >85 % purity was extracted from the peels of fresh lemons (*Citrus limon*). Eriocitrin doses, 10, 25 and 100 mg/kg of body weight (bw)^(22–25)^, were homogenised in the HFD to mimic a natural intake by the food. The following procedure was used to avoid losses by food intake fluctuations: The mice food intake was measured daily, and then, the measured food intake of each mouse was used to prepare the next day HFD containing the determined doses of eriocitrin. The food of next day was offered in 110 % of the earlier day food intake, being adjusted according to their intake. Then, to assure the intake of eriocitrin in the right doses, the dose of each group was added to the HFD to have 120 % of the determined dose. Thus, if mice consumed only 100 % of the food offered, they would still have consumed 110 % of the determined dose, if the mice consumed 90 %, they would still have consumed around 100 % of the dose.

### Diets

The HFD (Rhoster, Indústria e Comercio LTDA, Araçoiaba da Serra, SP, Brazil) was prepared with purified ingredients to contain 45 % kcal from fat (lard), 21 % kcal from protein (casein) and 34 % kcal from carbohydrates (28 % corn starch and 72 % sucrose), with a caloric density of 5⋅4 kcal/g. The standard diet was based on AIN-93M and contained 9⋅5 % kcal from fat (soybean oil), 14⋅7 % kcal from protein (casein) and 75⋅8 % kcal from carbohydrates (82 % corn starch and 18 % sucrose), with a caloric density of 4⋅2 kcal/g^(26)^. The composition of the diets is shown in Table 1.

**Table 1.** Composition of high-fat diet and standard diet

Diets	High fat	Standard
*Ingredients (g/kg)*		
Corn starch	78	466
Maltodextrin	117	155
Sucrose	201	100
Casein	240	140
L-cisteine	3⋅5	1⋅8
Soybean oil	29	40
Lard	207	0
Fibre	58	50
Vitamin mix	12	10
Mineral mix	12	35
Dibasic calcium phosphate	15	0
Calcium carbonate	6⋅4	0
Potassium citrate	19	0
Choline bitartrate	2⋅3	2⋅5
Tert-butylhydroquinone	0⋅1	8
*Composition (% kcal)*		
Protein	20⋅8	14⋅7
Carbohydrate	33⋅8	75⋅8
Fat	45⋅4	9⋅5
*Energy (kcal/g)*	5⋅4	4⋅2

### Sample collection

Mice were fasted overnight for a total 10 h, anaesthetized with xylazine (16 mg/kg): ketamine (60 mg/kg) *via* i.p. for euthanasia by cardiac puncture and blood collection. Blood was collected into tubes with separating gel and clot activator, and centrifuged at 2800 rpm for 15 min for blood serum recovery. Abdominal adipose tissue (retro-abdominal, epididymal and perirenal), liver, spleen, heart and kidneys were collected after frontal excision, rinsed with 0⋅9 % saline, blotted in filter paper, weighed and immediately frozen in liquid nitrogen. Blood serum and organs were stored at −80°C until analysis and liver left lobes were reserved for histological analysis.

### Histological analysis

Liver tissues were kept in 10 % buffered formalin for 48 h, then rinsed with deionised water and stored in 70 % ethanol until analysis. Fixed tissues were paraffin-embedded, sectioned at 4 μm and stained with hematoxylin-eosin. The results of staining were submitted for evaluation in an optical microscope with a magnifying power of 200×^(27)^ by using standard protocols from The Pathology Laboratory from The São Paulo State University (UNESP), School of Dentistry, Araraquara, SP, Brazil. Hepatic morphology, fat deposition and cell damage were assessed by an expert pathologist in a blind fashion.

### Blood and liver metabolic parameters

Commercial enzymatic assay kits (Labtest, Lagoa Santa, MG, Brazil) were used to measure blood serum glucose, total-cholesterol, HDL-cholesterol, triacylglycerols, alanine and aspartate aminotransferases (ALT and AST), and NEFA (non-esterified fatty acid) (Cayman Chemical, Ann Arbor, MI, USA). LDL-cholesterol concentration was calculated by Friedewald's formula: LDL = (CT − HDL) − (TG/5). Insulin was assayed by ELISA Multiplex kits using a Luminexx MAP detection method (Merck KGaA, Darmstadt, Germany). Hepatic lipids were extracted with chloroform: methanol (2:1), dried under N_2_ at −60°C and solubilised with Triton X-100 according to Folch's method^(28)^ and the triacylglycerols and total-cholesterol were determined using the same tests used for blood lipids, and expressed as milligram/gram of liver tissue. Fasting blood glucose and insulin concentrations were used to estimate insulin resistance by The Homeostatic Model Assessment (HOMA) index, as: HOMA-IR = Insulin (μU/ml) × glucose (mmol/l)/22⋅5^(29)^.

### Inflammatory markers

Inflammatory cytokines (TNF-α, IL-1β, IL-6, IL-10, MCP-1, adiponectin, resistin and leptin) were assayed by ELISA Multiplex kits using a Luminexx MAP detection method (Merck KGaA, Darmstadt, Germany) and us-CRP by immunoturbidimetry (Labtest, Lagoa Santa, MG, Brazil).

### Oxidative stress markers

The thiobarbituric acid-reactive substances method (TBARS) was used to determine blood serum lipid peroxidation as previously described^(30)^. TBARS levels were spectrophotometrically measured at 535 nm and expressed as μM MDA. Total antioxidant activity was determined by the ABTS assay as previously described^(31)^. Formation of ABTS^•+^ radical was measured at 734 nm, using Trolox (Sigma-Aldrich^®^, St. Louis, MO, USA) as standard. Values were expressed as mM of Trolox equivalent antioxidant capacity (mMeq TEAC). All analyses were performed in triplicate.

### Statistical analysis

The effects of eriocitrin and/or obesity on the studied groups were analysed by one-way ANOVA. The sample size was established using Sigma Stat 3.11, type I error α = 0⋅05 and type II β error = 0⋅15 (85 % power), based on a previous study^(32)^, where the reduction of fasting glycaemia was observed after 4 weeks of treatment with hesperidin. The estimated minimum sample size was ten individuals per group, and fifty animals were allocated into five groups using simple random sampling. When there was a significant difference between the groups, the Tukey's *post hoc* test was performed at a significance level of 5 % (*P* ≤ 0⋅05) at GraphPad Prism 6 software. Results are presented as mean ± standard deviation.

## Results

### Food and eriocitrin intake, body weight and organs

**Fig. 1.**
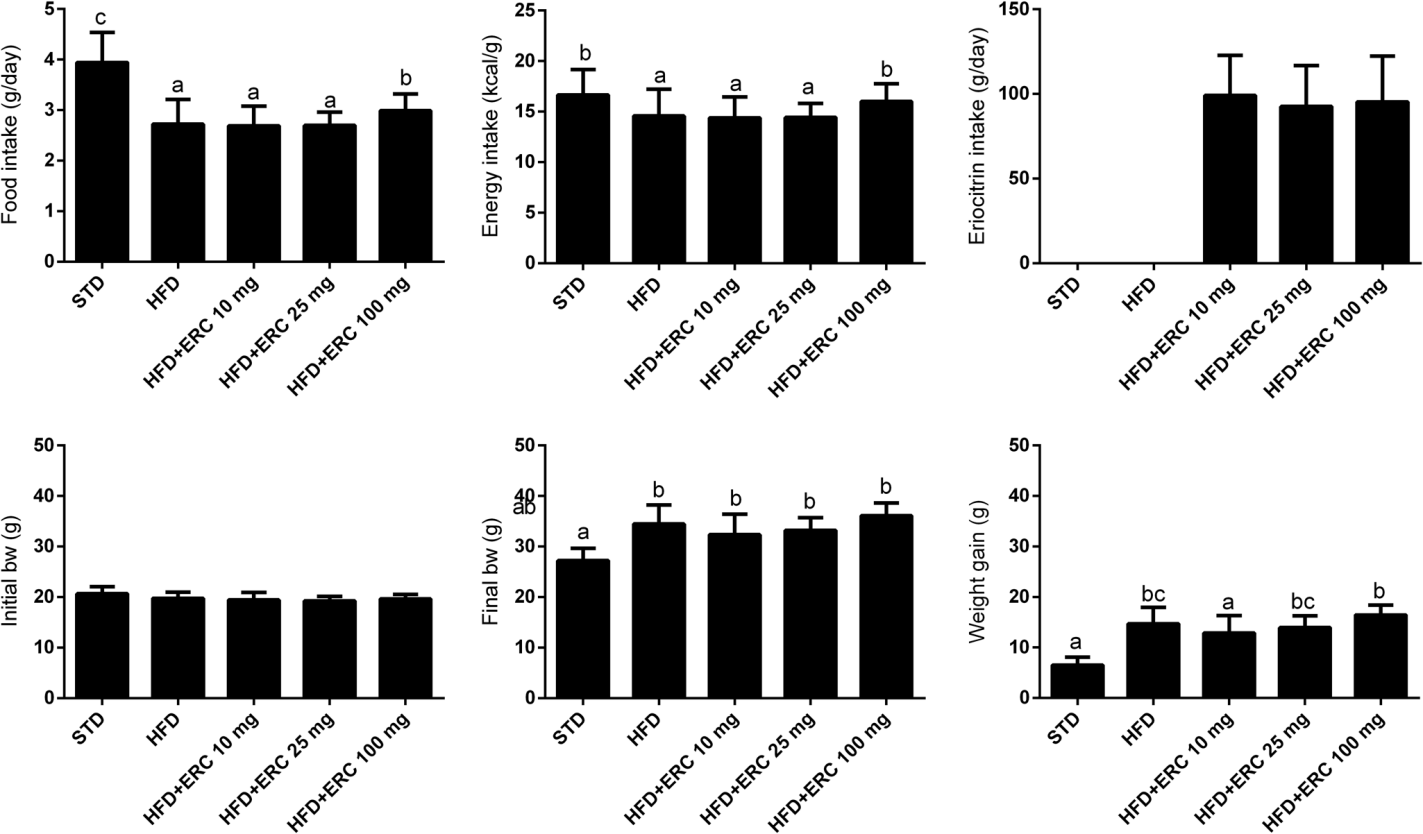
Food and energy intake, and body weights of C57BL/6J male mice fed standard diet (STD), high-fat diet (HFD) and HFD supplemented with different doses of eriocitrin (ERC). Values are means ± sd. Different letters mark statistically significant differences, *P* < 0⋅05.

**Fig. 2.**
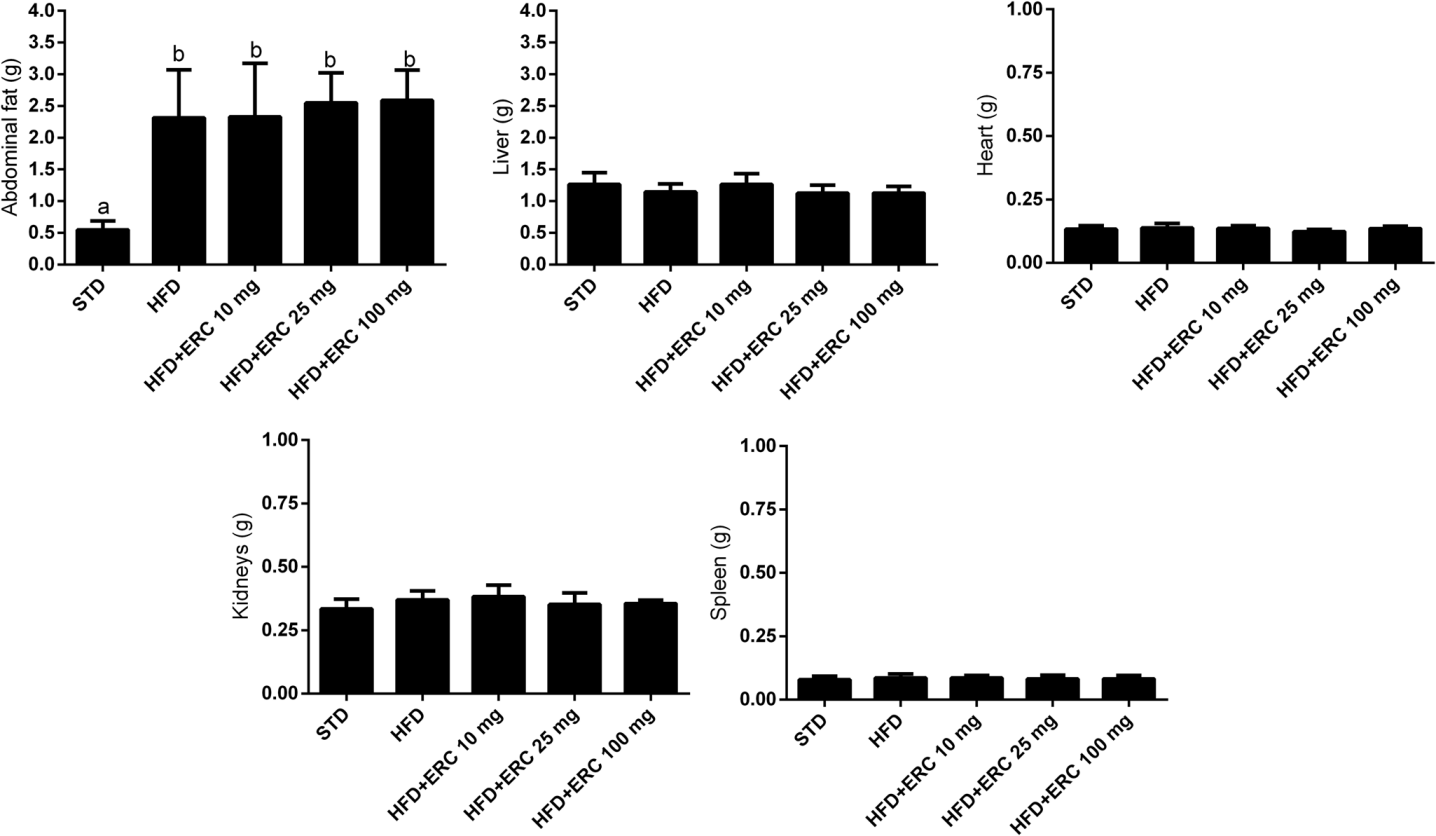
Organ weights of C57BL/6J male mice fed standard diet (STD), high-fat diet (HFD) and HFD supplemented with different doses of eriocitrin (ERC). Values are means ± sd. Different letters mark statistically significant differences, *P* < 0⋅05.

Mice fed an HFD, with or without eriocitrin supplementation, increased body weight by 25 % and gained weight by 123 % compared to the STD group (*P* < 0⋅05) (Fig. 1). Although the HFD groups had an average abdominal fat accumulation 4⋅4 times higher than controls (*P* < 0⋅05), their food and energy intake were lower than the control group (Fig. 2). The greatest gains in weight, food and energy intake were from the group supplemented with 100 mg/kg bw eriocitrin. No change in liver, heart, spleen and kidney weight was observed between HFD-fed mice and control animals (Fig. 2). The estimated dose of eriocitrin by the mean daily intake of food was on average 95⋅6 ± 24⋅9 % of the expected dose of eriocitrin in all supplemented groups.

### Histological analysis

**Fig. 3.**
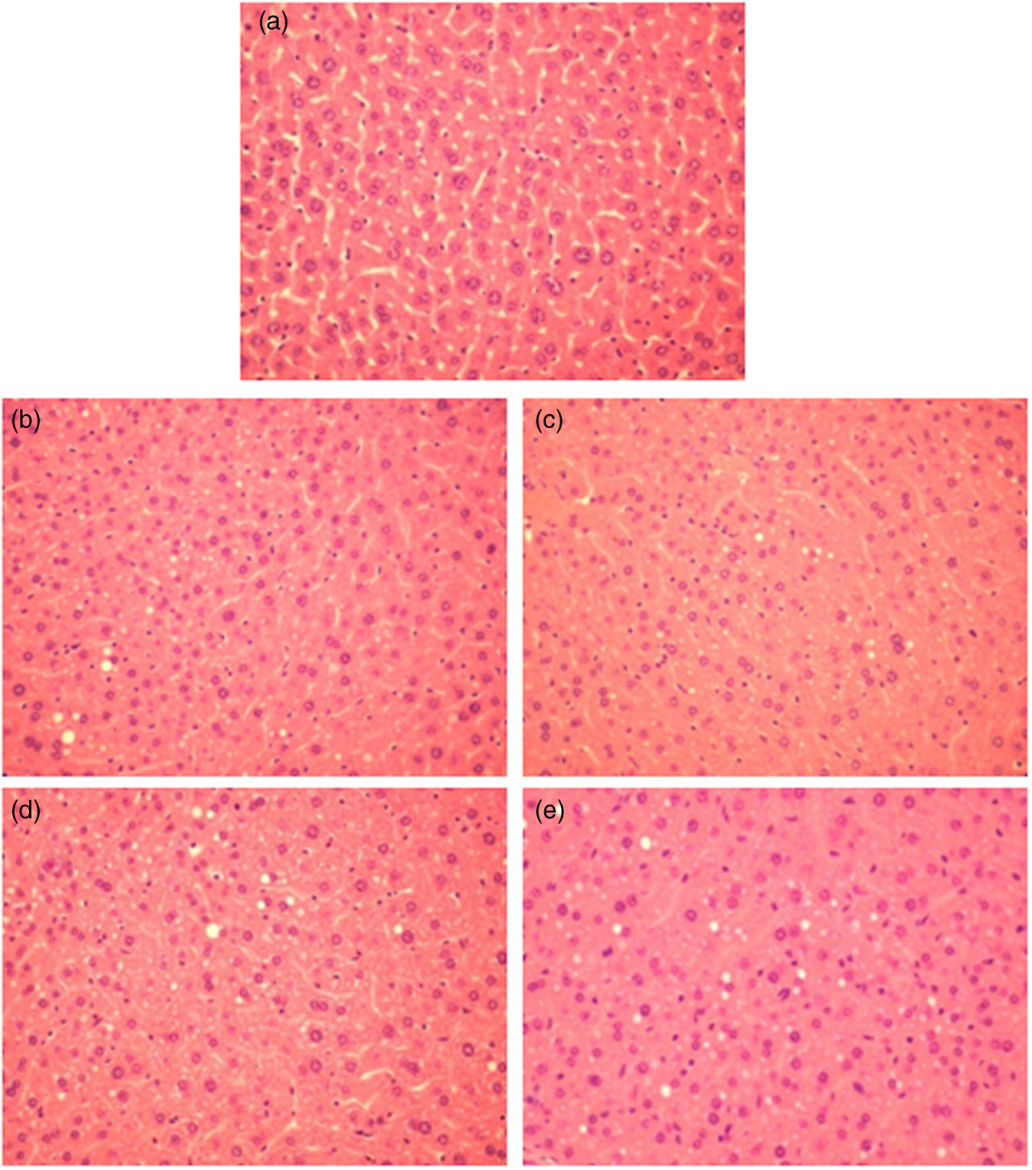
Histological sections of C57BL/6J male mice fed standard diet (STD) (a), high-fat diet (HFD) (b), high-fat diet (HFD) supplemented 10 mg/kg bw of eriocitrin (ERC) (c); HFD supplemented 25 mg/kg bw of ERC (d); and HFD supplemented 100 mg/kg bw of ERC (e). (a) displays well-defined contours, globular cells, and micro, almost not visible, lipid droplets, while apparent lipid droplets, undefined contours and bigger cells are observed in (b) (the same pattern is seen in c, d and e).

The morphological findings of liver sections of mice fed standard diet were histologically normal, exhibiting globular hepatocytes with one or two centralised nuclei and apparent basophilic nucleoli. The cytoplasm was eosinophilic and did not present substantial macro- or microvesicular fat vesicles (Fig. 3(a)). On the other hand, livers from mice fed HFD exhibited the classical hepatic steatosis, with varying sizes of fat vesicles in distinct areas of the tissue, characterising micro- and macrovesicular steatosis (Fig. 3(b)). Their hepatocytes were larger compared to those of the control group, and many lacked well-defined contours, and/or were fragmented with undefined nuclei and nucleoli. However, no infiltration by leukocytes was observed in the hepatic parenchyma, and the portal space region was within normal expectations. Livers from mice supplemented with eriocitrin exhibited characteristics similar to those found in the non-supplemented HFD-fed mice (Fig. 3(c)–(e)).

### Metabolic parameters

**Fig. 4.**
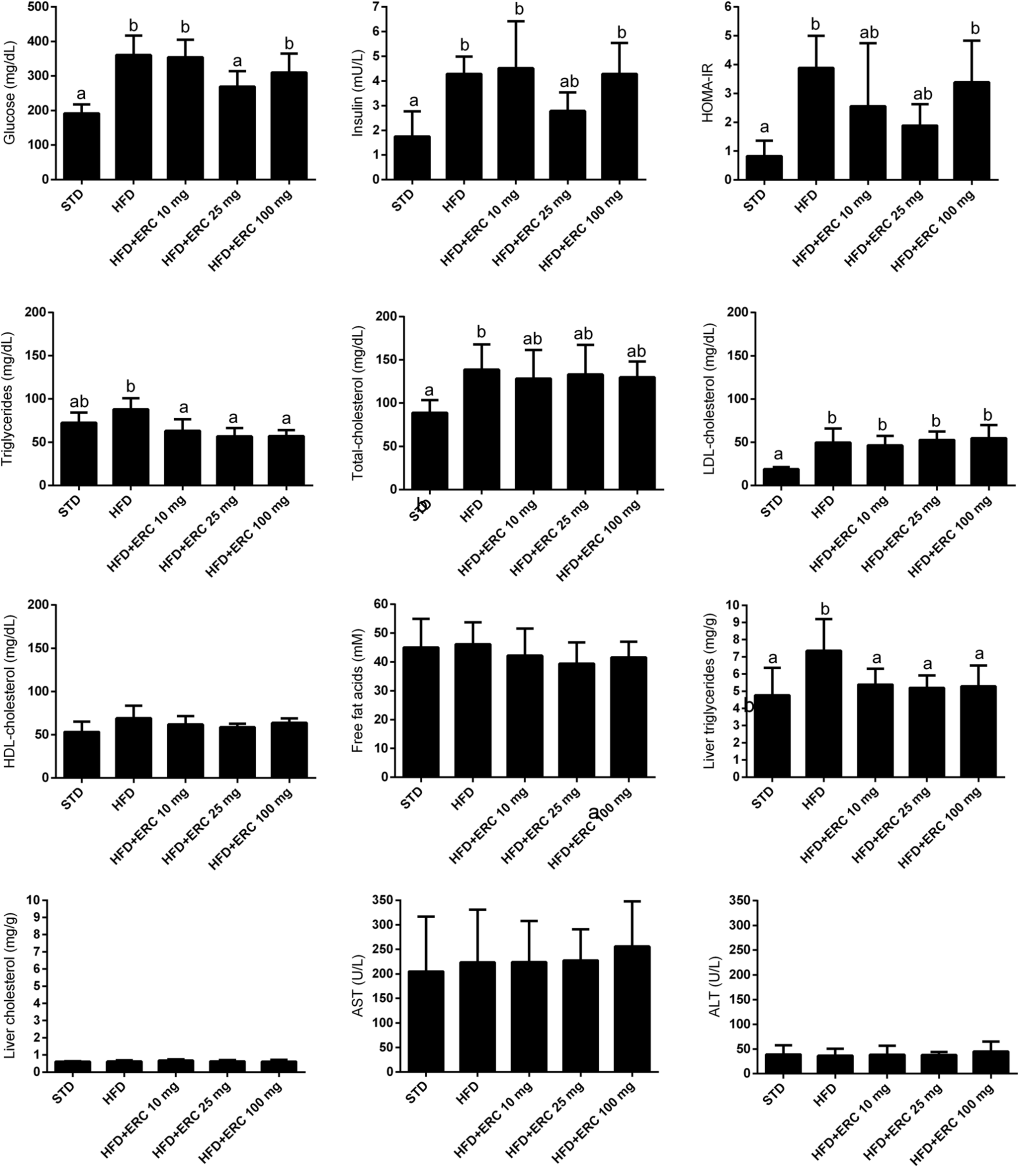
Blood and liver metabolic parameters of C57BL/6J male mice fed standard diet (STD), high-fat diet (HFD) and HFD supplemented with different doses of eriocitrin (ERC). Values are means ± sd. Different letters mark statistically significant differences, *P* < 0⋅05.

Compared to the STD group, mice induced to obesity by the HFD exhibited higher levels of blood serum glucose and insulin, with a consequentially higher insulin resistance index HOMA-IR. On the other hand, the group supplemented with 25 mg/kg bw eriocitrin exhibited significantly lower levels of blood serum glucose and intermediate levels of insulin. The insulin resistance index values showed moderate decreases for both the groups supplemented with 10 and 25 mg/kg bw eriocitrin (Fig. 4).

Blood serum triacylglycerols concentrations increased in the HFD group; however, the eriocitrin supplemented groups, irrespective of the dose, exhibited significantly lower concentrations of serum triacylglycerols, with values lower than the control group. Accordingly, hepatic triacylglycerol increased in the HFD group, while the groups supplemented with eriocitrin kept values similar to those of the control group (Fig. 4). Total-cholesterol in blood serum increased significantly after feeding on the HFD, while tall of the groups supplemented with eriocitrin showed intermediary levels. No significant differences were observed in liver total-cholesterol (Fig. 4). Likewise, serum LDL-cholesterol increased in all groups fed the HFD and was not altered by eriocitrin at the doses tested. Blood serum HDL-cholesterol, NEFA, ALT and AST were not changed (Fig. 4).

### Inflammatory and oxidative stress parameters

**Fig. 5.**
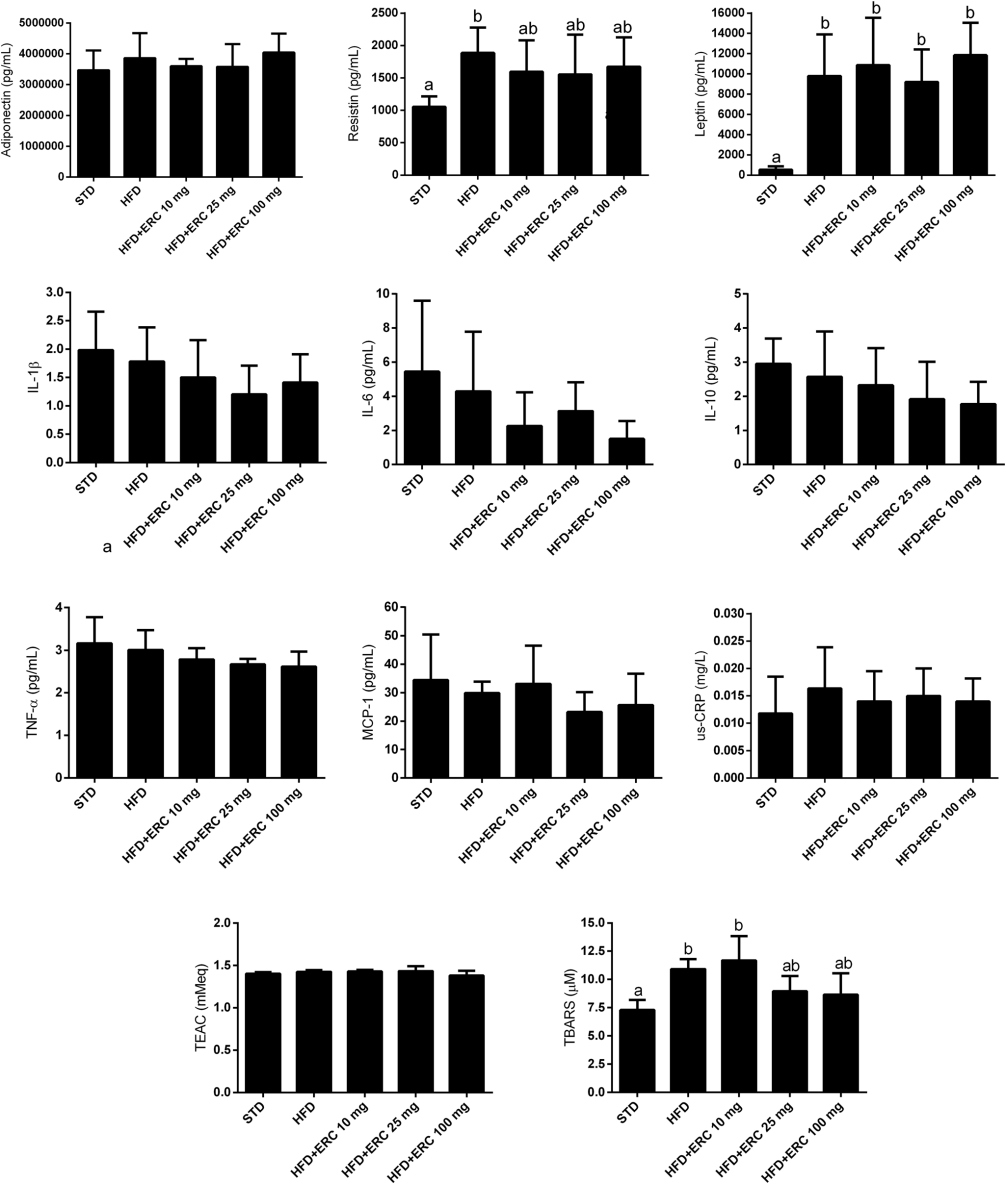
Inflammatory and oxidative stress parameters of C57BL/6J male mice fed standard diet (STD), high-fat diet (HFD) and HFD supplemented with different doses of eriocitrin (ERC). Values are means ± sd. Different letters mark statistically significant differences, *P* < 0⋅05.

The adipokines, resistin and leptin were increased in mice induced to obesity by the HFD, while eriocitrin at 10, 25 and 100 mg/kg was able to moderately reduce resistin, but not leptin. Systemic levels of adiponectin and the other inflammatory markers, including IL1β, TNF-α, MCP-1 and us-CRP were not affected by HFD or eriocitrin supplementation (Fig. 5). But, there appeared to be a trend to lower IL-6 values in the mice fed the HFD supplemented with the three dose levels of eriocitrin.

The lipid peroxidation in blood serum increased in mice fed the HFD but was moderately lower in groups supplemented with 25 and 100 mg/kg of eriocitrin (Fig. 5). No significant changes were observed for the total antioxidant capacity (TEAC assay) in blood serum of mice fed HFD, supplemented or not (Fig. 5).

## Discussion

Dietary supplementation with eriocitrin at low (10 and 25 mg/kg bw) and high (100 mg/kg bw) doses improved the metabolic, inflammatory and oxidative stress parameters in HFD-induced obesity in mice. Eriocitrin supplementation reduced blood serum levels of triacylglycerols (−31 %), total-cholesterol (−6 %), and liver triacylglycerols (−28 %). Eriocitrin also improved lipid peroxidation (−19 % at 25 mg/kg and −22 % at 100 mg/kg), and markers of insulin resistance, such as resistin (−15 %) and insulin resistance index (−34 % at 25 mg/kg and −39 % at 100 mg/kg). Moreover, it significantly decreased serum glucose levels (−25 %) and improved levels of insulin (−35 %) of the group supplemented with the dose of 25 mg/kg.

As observed with high doses of eriocitrin (100 and 200 mg/kg)^(11)^, the present study shows that much lower doses (10 and 25 mg/kg) of eriocitrin are effective in improving lipid metabolism. The lower doses of eriocitrin reduced triacylglycerol levels in both liver and blood serum and improved the levels of serum total-cholesterol. However, the histological analysis was not able to show the reduction of fat deposition in the liver. In our previous study, short-course supplementation with eriocitrin did not prevent liver fat deposition in mice fed HFD for 4 weeks, but its aglycon eriodictyol did^(11)^. The better absorption of the aglycon form in relation to glycosylated forms of citrus flavonoids has been observed, and many researchers chose to evaluate the potential of the aglycons since this form has higher bioavailability^(33)^. Thus, in a shorter time course, supplementation with eriocitrin aglycon (eriodictyol) was able to prevent liver fat accumulation promoted by HFD, while eriocitrin had no effect, probably due to the higher bioavailability of its aglycon. Previous studies showed that eriocitrin may reduce serum and liver triacylglycerols through increased β-oxidation in the liver and white adipose tissue, and that eriocitrin effects on serum total-cholesterol may be mediated by the increased excretion of bile acid together with the induction of LDL receptor, and the inhibition of HMG-CoA reductase in the liver^(15,34,35)^. In zebrafish supplemented with eriocitrin, the reduction of liver fat deposition was indicated as a response to the increased mitochondrial biogenesis and ATP production^(15)^. Also, the supplementation of mice fed an HFD with lemon polyphenols (mostly composed by eriocitrin and hesperidin) was shown to increase peroxisomal β-oxidation and suppress body weight gain and body fat accumulation due to higher levels of acyl-CoA oxidase mRNA in the liver and white adipose tissue, through positive regulation of the mRNA level of PPARα^(35)^. In addition, mice supplemented with the lemon polyphenols extract showed lower levels of glucose, insulin and of the insulin resistance index (HOMA-IR), which were suggested as a response to the increased peroxisomal fatty acid β-oxidation, and therefore, the reduced-fat pad accumulation in the white adipose tissue^(35)^.

Although obesity is considered a low-grade systemic inflammatory state, in the present study, we did not observe differences in us-CRP and in most of the cytokines analysed in blood serum (IL-6, TNF-α, IL-1β, IL-10, MCP-1 and leptin). However, the level of resistin, a cytokine produced in the adipose tissue, was moderately lowered by eriocitrin, and there were trends towards lower values of IL-6 and MCP-1, which could in part explain the effects of eriocitrin in the improvement of lipid and glucose parameters. Resistin is well known to decrease insulin sensitivity and enhance adipocyte inflammation, contributing to the lipid dysfunction in obesity. A preliminary study showed that resistin administration impaired glucose tolerance and insulin action in healthy mice, while anti-resistin antibodies could improve blood sugar and insulin sensitivity in mice with diet-induced obesity^(36)^. In our previous study, mice fed HFD for 4 weeks, half that of the present study, showed elevated levels of us-CRP, IL-6, TNF-α and MCP-1 in the blood serum, while supplementation with 200 mg/kg eriocitrin completely inhibited their increase^(11)^. It has been shown that the elevation of inflammatory cytokines and changes in acute-phase proteins in mice fed HFDs are a rapid and transient response that is developed at two distinct stages, the first in the fourth week and the second between the 12th and 16th weeks, when the inflammation in white adipose and muscle tissues becomes apparent, and that between these times the inflammation may not be detected^(37)^.

Besides, reduction of oxidative stress can attenuate adipose tissue inflammation and reduce insulin resistance, hyperlipidemia and hepatic steatosis^(38)^. In this sense, eriocitrin was shown to reduce markers of lipid peroxidation and DNA damage present in the liver and kidneys of diabetic rats^(39)^. The antioxidant action of eriocitrin occurs directly by the uptake of oxygen radicals and the modulation of cell signalling pathways promoting the activation of endogenous defense mechanisms^(40–42)^. In the present study, the effect of eriocitrin on lipid peroxidation was observed at doses of 25 and 100 mg/kg, but not at the dose of 10 mg/kg, suggesting a minimum dose of 25 mg/kg to exert its antioxidant activity. Although not all doses of eriocitrin were capable to affect lipid and glucose metabolism, the improvement of insulin resistance observed in this study is suggested to be a sum of the antioxidant activity of eriocitrin and its effects on lipid metabolism. This may represent an important pathway by which eriocitrin protects against risk factors associated with metabolic syndrome and obesity.

Of the three doses tested, the dose of 25 mg/kg bw showed the best metabolic, anti-inflammatory and antioxidative response in the parameters analysed. Of particular, strength was the effects on glucose and lipid metabolism, especially the reduction of blood glucose, and of the blood and liver triacylglycerols, and the improvement of serum levels of lipid peroxidation and resistin. Therefore, our results showed that low doses of dietary eriocitrin are able to counteract the deleterious effects of HFD and prevent risk factors of metabolic syndrome and chronic diseases related to obesity. Further, the use of lower doses may help to prevent unintended complications possibly occurring at much higher doses of potent antioxidant supplements such as eriocitrin^(43)^.
